# Serum Anti-HPV Antibody Titer as a Marker of Vaccine Effectiveness in Males with Genital Infection

**DOI:** 10.3390/vaccines8040743

**Published:** 2020-12-07

**Authors:** Luca De Toni, Francesco Muscianisi, Christian Corsini, Marco Ghezzi, Andrea Di Nisio, Carlo Foresta, Andrea Garolla

**Affiliations:** Unit of Andrology and Reproductive Medicine, Section of Endocrinology, Department of Medicine, Centre for Male Gamete Cryopreservation, University of Padova, 35122 Padova, Italy; luca.detoni@unipd.it (L.D.T.); francesco.muscianisi7@gmail.com (F.M.); corso.chri@gmail.com (C.C.); ghezdoc@hotmail.it (M.G.); andrea.dinisio@unipd.it (A.D.N.); carlo.foresta@unipd.it (C.F.)

**Keywords:** HPV-adjuvant vaccination, male infertility, serum antibodies, warts, fluorescence in situ hybridization

## Abstract

*Introduction:* Persistent human papillomavirus (HPV) semen infection is increasingly associated with male infertility. Adjuvant HPV vaccination is suggested to reduce the time to clearance and the disease relapse in males with persistent HPV semen infection. However, only a sub-population of patients show a clinical benefit from adjuvant vaccination. Here, we aimed to address the effectiveness rate of HPV adjuvant vaccination in males with genital tract infection and the possible prognostic markers of healing. *Methods:* Clinical records from 379 patients with persistent seminal HPV detection, all receiving HPV adjuvant vaccination, were considered. Clinical data, including genital HPV-DNA assessment by INNO-LiPA genotyping, semen HPV-DNA analysis by FISH analysis and serum antibody titer, were collected at basal (T0) and after 6 months (T1) since the vaccination cycle ended. *Results:* Clearance of genital HPV-DNA was recorded in 326 (86%) patients. Serum HPV-antibody titer at T1 was the most important prognostic factor associated with HPV-DNA clearance. A serum antibody titer equal to or greater than the threshold value 1:125, obtained by ROC curve analysis, was prognostic of healing. *Conclusions:* Anti-HPV antibody represents a suitable marker of adequate immune response to HPV vaccination in patients with genital infection.

## 1. Introduction

Human Papillomavirus (HPV) is the etiological agent of the most common sexually transmitted infection worldwide, with an estimated 6.2 million new cases annually [1]. The reported prevalence of the infection in the whole male population, according to a recent meta-analytical estimate, is 49%, and ranges from condylomas to epithelial tumors, such as penile, anal and oro-pharyngeal carcinomas [2]. In addition to male genital areas, an increasing number of studies reported the detection of HPV virions in whole semen and even bound to the sperm cells [3,4]. This condition has also been associated with worsened sperm motility and higher incidence of anti-sperm antibodies (ASAs) compared to fertile men, suggesting a major role of HPV infection in male infertility [4,5,6]. Finally, recent findings show that HPV virions bound to sperm represent a negative prognostic factor for natural and assisted reproductive outcome [7].

HPV vaccination is usually administered before the beginning of sexual activity in a three-dose schedule at 0, 2 and 6 months. However, recent evidence showed the efficiency of a single dose of vaccine administered to females younger than 18 years old [8]. Of note, HPV quadrivalent vaccination is considered an efficient and safe approach to prevent HPV infection and related diseases induced by vaccinal genotypes (6, 11, 16, 18) through the likely increase of the levels of mucosal neutralizing antibodies [9,10]. Moreover, HPV vaccines offer cross-protection against a few specific non-vaccine HPV types for individuals without previous infection, with the quadrivalent vaccine showing some degree of protection against HPV 31, 33, and 45, for persistent infection and CIN2+disease, and little evidence of cross-protection against HPV 52 and 58 [11]. On the other hand, some authors suggested that vaccination administered in patients with HPV-related carcinomas (especially head and neck cancers) showed a lower rate of relapse after lesions removal [12]. Also, del Pino et al. demonstrated a significant reduction of HPV infection persistency or recurrence after HPV vaccination applied to women undergoing conization [13]. Moreover, adult males with persistent HPV detection in semen had reduced time to clearance after vaccination compared to infected controls, who did not undergo vaccination [14]. However, as previously shown in subjects with HPV ano-genital tract infection, vaccination is efficient in shortening HPV time to clearance from semen only in a subset of infected males, despite the fact that seroconversion is achieved in all infected patients after vaccination [15].

Currently, there is no validated treatment for asymptomatic patients with HPV infection. However, it is widely acknowledged that the development of a specific humoral immune response, whether natural or obtained by vaccination, is the only condition able to counteract reinfection and disease recurrences [16]. Recent studies showed that vaccination is effective only in a subset of infected patients, suggesting further widening of this topic [15]. The aim of this study was to address the association between HPV adjuvant vaccination and the clearance of HPV infection from the genital tract. In addition, we aimed to identify possible prognostic markers of healing.

## 2. Materials and Methods

### 2.1. Patients 

The study was conducted at the Unit of Andrology and Reproductive Medicine of the University Hospital of Padova, according to the principles of the Declaration of Helsinki, and approved by the Institutional Ethics Committee of the Padua General Hospital by Protocol No. 2336P and subsequent amendments. Among males attending our unit for andrological issues (genital warts and/or infertility associated with risk factors for HPV infection such us previous warts, HPV-infected partner, idiopathic asthenozoospermia, anti-sperm antibodies), we retrospectively considered the clinical and demographic records from 700 patients with genital and/or semen HPV detection and genotyping. Inclusion criteria were: acceptance of written informed consent for clinical data access, persistent HPV infection (at least 6 months) from genotypes included in the quadrivalent vaccine, such us 6, 11, 16, 18, or those with evidence of cross-protection, such as 31, 33, 45, 52 and 58 [11] and patients receiving HPV vaccination (3 doses of quadrivalent vaccine, administered respectively at study initiation, after 2 months and after 6 months from first dose). The quadrivalent vaccine Gardasil (Merck Serono S.p.A., Milano, Italy) was administered off-label, as previously described [14]. Exclusion criteria were, presence of any other concomitant sexually transmitted infections of the semen (*Chlamydia trachomatis*, *Neisseria gonorrhoeae*, *Mycoplasma* spp.), Human Immunodeficiency Virus (HIV), Hepatitis B Virus (HBV), Hepatitis C Virus (HCV) and *Treponema pallidum* active infections, CFTR gene mutations and any alteration of the major endocrine axes.

The analysis was performed on data collected at study initiation (T0) and after 6 months from the end of the vaccination cycle (T1), thus one year since the study initiation, in a final sample size of 379 patients fulfilling inclusion criteria. Vaccine responding was defined as the absence of HPV-DNA in seminal fluid and cells coming from balano-preputial sulcus swab, detected by INNO-LiPA Genotyping assay, at T1.

### 2.2. Semen Analysis

Semen analysis and anti-sperm antibodies were assessed according to the World Health Organization (WHO) Criteria. HPV-DNA bound to sperm was detected by FISH analysis (FISH-HPV) on whole semen, as previously described [4].

### 2.3. HPV Detection And Genotyping

HPV-DNA detection and genotyping were performed on balano-preputial sulcus swab (Copan Italia S.p.A., Brescia, Italy) and in semen samples by INNO-LiPA Genotyping Extra assay, as previously described [4]. This analysis allowed the identification of the following HPV types: 6, 11, 16, 18, 26, 31, 33, 35, 39, 40, 42, 43, 44, 45, 51, 52, 53, 54, 56, 58, 59, 66, 68, 69, 71, 70, 73, 74 and 82.

### 2.4. Anti-HPV Antibodies

The detection and titer of serum total anti-HPV immunoglobulin G (IgG) were assessed on serum samples from peripheral blood obtained from all patients, at T0 and T1, by enzyme linked immuno-sorbent assay (ELISA) using a commercial kit supplied by DRG Diagnostic GmbH (Marburg, Germany), as previously described [14]. An international standard for antibodies to HPV 16 was available only from 2011 onwards, making it impossible to determine the absolute titer samples collected previously [17]. Accordingly, the serum antibody titer was determined by serially diluting samples (1:10, 1:100 and 1:1000, respectively) that were assessed according to the manufacturer’s instructions. The antibody titer was then defined as the lowest dilution displaying an optical density (O.D.) equal to or lower than a cut-off value given by the mean O.D. + 2 × standard deviation (SD) of a negative control [18].

### 2.5. Statistical Analysis

Statistical analysis was performed using SPSS version 23.0 (SPSS Inc., Chicago, IL, USA). Data are presented as means ± standard error of the mean (SEM) for continuous variables and as percentages for categorical variables. Comparison of continuous variables between two groups, particularly demographic data, semen parameters and antibody titers between healed and not-healed patients, was performed by Student’s t-test for unpaired data, previously assessed for normal data distribution by the Kolmogorov–Smirnov test. Proportions and discrete variables, when the number of groups was <5, were compared by the Fisher’s chi-squared exact test. *p*-values < 0.05 were considered as statistically significant. The possible relevance of prognostic factors was assessed by calculating the odds ratio (OR) and related 95% confidence intervals (95% CI).

The receiver operating characteristic (ROC) curve was calculated to correlate the antibody titer recorded at T1 with the healing rate after vaccine administration. The area under the curve (AUC) thus determined was assessed through the classification of Swets. The threshold value of the anti-HPV antibody titer for healing was calculated with Youden’s S statistic. For the considered comparison, 0.9 < AUC < 1.0 was found. The multivariate analysis through logistic-stepwise regression was also applied to continuous and non-continuous variables in order to evaluate the association with vaccine responding as a dichotomous outcome.

## 3. Results

Clinical and demographic data of the study group are reported in Table 1. At the follow-up visit at T1, 326 out of 379 patients receiving vaccination (86%) showed the complete clearance of HPV-DNA, assessed by the INNO-LiPA Genotyping assay, at both the seminal and genital level, and were therefore considered responding. Interestingly, responding patients had a lower prevalence of concomitant drug therapy assumption and genital warts.

Table 2 shows the comparison of semen and serum data obtained at T0 and T1. A reduction of both the percentage of patients showing positive FISH-HPV and ASA, between T1 and T0, was observed in the whole population (respectively: 43.8% vs. 7.1%, *p* = 0.0001 for FISH-HPV and 23.1% vs. 14.2%, *p* = 0.0021 for ASA). In particular, this evidence was essentially associated with the significant reduction of these two parameters in responding patients (*p* < 0.001 FISH-HPV) and *p* = 0.0015 ASA, respectively. In fact, such a significance for FISH-HPV and ASA was not observed in non-responding patients (both *p* > 0.05).

Vaccination was associated with seroconversion in all patients, with an overall increase of the mean serum-antibody titer from 1:72 ± 36.47 at T0 to 1:308 ± 42 at T1 (*p* = 0.0001). The significant increase of serum-antibody titer at T1 vs. T0 was also maintained when patients were distinguished into responding and non-responding. Importantly, in responding patients, the serum-antibody titer at T1 was higher than that observed in non-responding patients at the follow-up (respectively: 1:469 ± 58 vs. 1:62 ± 7; *p* = 0.0001).

**Figure 1:**
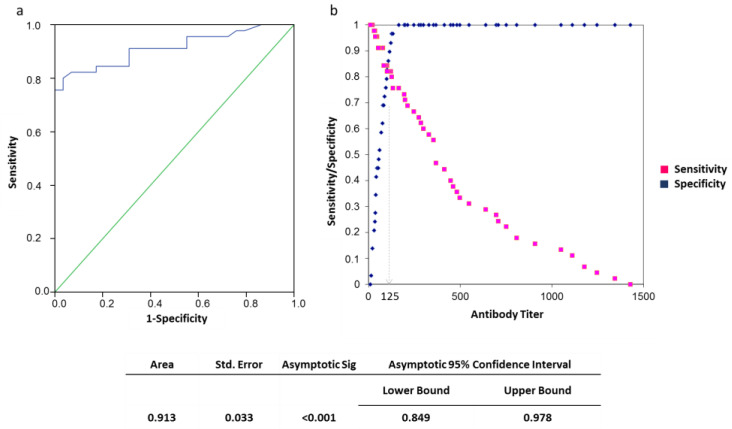
(**a**) Receiver operator characteristics (ROC) curve for antibody titer against healing after 6 months from vaccination cycle end. (**b**) In the graph, sensitivity (red dots) and specificity (blue dots) are plotted against the antibody titer value. The maximum value of Youden index is reported (dotted line).

In this regard, the application of the ROC curve-analysis to test sensitivity and specificity of the HPV-antibody titer assessment showed that a serum titer equal to or greater than the cut-off value of 1:125 was prognostic of healing (OR 1.78; 95% CI: 1.17–2.70; Figure 1).

Of note, the serum HPV-antibody titer at T1 was the factor most significantly associated with the responding outcome in a multivariate analysis through the logistic-stepwise regression approach (Score = 4.984; *p* = 0.026).

## 4. Discussion

Male subjects presenting with HPV genital infection have an acknowledged longer persistence than that observed in females [19]. This evidence has been associated with a lower and delayed humoral response to HPV in males [20]. Recent evidence supports the clinical effectiveness of vaccination in patients with HPV-related disease, particularly in males with genital infection in which vaccination nearly halves the time to viral clearance compared to naïve patients [14,15]. However, this practice appears to be effective in 47–67% of cases with anogenital infection, regardless of the HPV status at enrolment [15]. In the present study, we showed that the effectiveness of HPV vaccination towards vaccine and cross-reactive genotypes is 86%, despite the fact that seroconversion was observed in all patients. In addition, we provided evidence that the absence of genital warts, the negative detection of HPV-DNA in semen and the absence of anti-sperm antibodies are positively associated with the response to vaccination. This observation suggests that the immune response associated with vaccination is more effective in the presence of an underlying subclinical infection. Most importantly, the achievement of threshold value of the serum-antibody titer following vaccination showed a major association with the responding process, suggesting this parameter as a marker of proper anti-HPV immune response. In particular, a serum-antibody titer greater than 1:125 represented a cut-off threshold, distinguishing patients undergoing genital HPV complete clearance after 6 months from the completion of the vaccination cycle. To the best of our knowledge, this result represents an unprecedented finding on this topic.

On these bases, the monitoring of serum anti-HPV antibody titer assumes clinical value for three reasons: (i) to disclose the natural immune response associated with a previous exposure to HPV, (ii) to assess the immune response associated with vaccination in patients with accidental genital infection and (iii) to critically consider a further vaccine dose, in order to increase humoral immunity, in those patients showing persistence of HPV infection, in spite of a vaccination-related seroconversion. This approach certainly requires appropriate clinical validation but has valid precedents since, for example, it represents a shared procedure adopted in subjects with a low response to HBV vaccination. [21].

## 5. Conclusions

Despite the fact that more studies including proper control arms are required to draw final conclusions, here, we provided evidence that HPV vaccination is able to improve the clearance of HPV from the genital tract. Moreover, the assessment of serum anti-HPV antibody titer represents a suitable approach to monitor the effectiveness of the anti-HPV immune response.

## Figures and Tables

**Table 1:** Clinical and demographic characteristics of 379 male patients with genital Human Papillomavirus (HPV) infection who received quadrivalent HPV vaccination, at study initiation (T0). Data were stratified according to vaccine responding at 6 months after the end of the vaccination cycle (12 months since study initiation, T1). Abbreviations: Resp. = Responding; Non-Resp. = Non-Responding; SEM = Standard error of the mean; BMI = Body mass index. Significant *p*-Values are in bold.

	Comparison of Data at T0 (Study Initiation)			
Parameters	Whole Population N = 379	Resp. at T1 326/379 (86%)	Non-Resp. at T1 53/379 (14%)	*p*-Value (Resp. vs. Non-Resp.)
Mean age (years ± SEM)	40.3 ± 0.65	39.8 ± 0.48	41.5 ± 1.44	0.9999
BMI (kg/m^2^ ± SEM)	23.9 ± 0.2	24.1 ± 0.19	23.6 ± 0.41	0.3175
Concomitant drug therapy (n/%)	104/379 (27.4%)	82/326 (25%)	22/53 (41.5%)	**0.0194**
Genital Warts (n/%)	123/379 (32.4 %)	90/326 (27.6%)	33/53 (62.3%)	**0.0001**
HPV Genotypes				
Vaccine genotypes (n/%)	227/379 (59.9 %)	191/326 (58.6%)	36/53 (67.9%)	0.2281
Cross-reactive genotypes (n/%)	152/379 (40.1%)	135/326 (41.4%)	17/53 (32.1%)	0.2281

**Table 2:** Serum and semen parameters of 379 male patients with genital HPV infection who received quadrivalent HPV vaccination, at study initiation (T0) and 6 months after the end of the vaccination cycle (12 months since study initiation, T1). Abbreviations: Resp. = Responding; Non-Resp. = Non-Responding; SEM = Standard error of the mean; FISH = Fluorescent in-situ hybridization; ASAs = Anti-sperm antibodies; Significance * *p* < 0.05 vs. the corresponding value at T0.

	T0 (Study Initiation)				T1 (6 Months after Vaccination Cycle Ending—12 Months Since Study Initiation)			
Parameters	Whole Population	Resp.	Non-Resp.	*p*-Value (Resp. vs. Non-Resp.)	Whole Population	Resp.	Non-Resp.	*p*-Value (Resp. vs. Non-Resp.)
Semen FISH-HPV DNA + (n/%)	166/379 (43.8%)	129/326 (39.6%)	37/53 (69.8%)	**0.0001**	27/379 (7.1%) *	0 *	27/53 (50.9%)	**0.0001**
Semen ASA + (n/%)	88/379 (23.1%)	64/326 (19.6%)	24/53 (45.3%)	**0.0002**	54/379 (14.2%) *	36/326 (11.0%) *	18/53 (33.9%)	**0.0001**
Seroconversion rate (n/%)	146/379 (38.5%)	128/326 (39.3%)	18/53 (33.9%)	0.5434	379/379 (100%) *	326/326 (100%) *	53/53 (100%) *	1.0000
Serum-antibody titer (dilution ± SEM)	1:72 ± 36.47	1:97 ± 34.4	1:28 ± 7.28	0.4199	1:308 ± 42 *	1:469 ± 58 *	1:62 ± 7 *	**0.0001**
